# Normal electric field enhanced light-induced polarizations and magnetic detection of valley polarization in silicene

**DOI:** 10.1038/s41598-020-73138-5

**Published:** 2020-10-06

**Authors:** N. Shahabi, A. Phirouznia

**Affiliations:** 1 Department of Physics, Azarbaijan Shahid Madani University, Tabriz, 53714-161 Iran; 2 Condensed Matter Computational Research Lab, Azarbaijan Shahid Madani University, Tabriz, 53714-161 Iran

**Keywords:** Physics, Condensed-matter physics, Spintronics

## Abstract

The role of staggered potential on light-induced spin and pseudo-spin polarization has been investigated in silicene. It has been shown that non-equilibrium spin and pseudo-spin polarizations are emerged in silicene sheet by applying an external perpendicular electric field in the presence of circularly polarized light emission. This electric field results in pseudo-spin resolved states very close to the Dirac points therefore could be considered as a pseudomagnetic field. It has been shown that staggered potential induced spin-valley locking and pseudo-spin resolved bands are responsible for the enhancement of the spin and pseudo-spin polarizations. Meanwhile, spin-valley locking suggests a coexistence of both spin and valley polarizations with nearly identical (or at least proportional) population imbalance at low Fermi energies which could be employed for magnetic detection of the valley polarization. It has been shown that spin-valley locking results in the protection of the spin polarizations against the relaxations in elastic scattering regime. In addition, the results indicate that the pseudo-spin current can be generated by the circularly polarized light which could be explained by asymmetric light absorption of the states in *k*-space.

## Introduction

Linear dispersion relation in the vicinity of Fermi energy in graphene leads to semi-metallic behavior which is described by the massless Dirac theory^1^. Graphene shows a variety of outstanding electronic and optical properties which make it one of the best candidates of optoelectronic applications^2,3^.

Monolayer graphene-like materials have attracted considerable attention during last decade. They inherit their rich physics from their famous counterpart i.e. graphene. Analogous to graphene, the basic structure of graphene-like materials is honeycomb lattice. The physics of this group of two dimensional (2D) materials is described by the massive Dirac fermions theory which stems from rather large intrinsic spin-orbit coupling that the latter originates from structural buckling^4^. The large ionic radius of silicon and group IV elements of the periodic table, leads to this structural buckling^4^.

Another prominent feature of buckled structures is the tunable band gap that can be controlled by an external electric field^5^. Silicene, germanene and stanene illustrate many attractive electronic and spintronic properties. Biased single layer silicene and germanene were reported to work effectively as field effect transistors while a vertical electric field can open a band gap in their semi-metallic band structure^6^.

Edge manipulation of the mentioned 2D materials were studied which can suggest very promising applications: a giant magnetoresistance which can lead to a “topological quantum transistor” and a perfect spin filter^7^. Potential application of silicene, germanene and stanene for Na or Li ion storage in Na or Li batteries has also been investigated^8^.

Any two-component quantum degree of freedom which is mathematically equivalent to spin, can be considered as ‘pseudo-spin’. In 2D systems with two sublattices, pseudo-spin portraits the sublattice degree of freedom with the eigenstates localized on *A* or *B* sublattices^9^. Pseudo-spin is analogous to the true electron spin but behaves totally different under time-reversal and parity inversion^10^. Pseudo-spin stems from the degeneracy between two inequivalent atomic sites per unitcell^11^. Meanwhile, Mecklenburg et al have demonstrated that the sublattice state vector represents indeed a real angular momentum in $3+1$ dimensions^11^.

Pseudo-spin is not associated with the internal magnetic moment of electron and does not interact with an external magnetic field however, it can turn into measurable orbital angular momentum^12^ and can manifest itself as an observable quantity and can be detected in transport phenomena and inter-band optical absorption^10^. Trushin et al have shown that due to the out-of-plane orientation of pseudo-spin, switching the helicity of circularly polarized light can cause a reduction or enhancement in inter-band absorption because the elements of inter-band transition matrix are sensitive to the light polarization and also pseudo-spin orientation in the initial and final states^10^. Although the pseudo-spin eigenstates are really robust, the exchange electron-electron interaction can alter the pseudo-spin orientation^10^.

In this work, light-induced non-equilibrium pseudo-spin polarization has been studied. Results show that pseudo-spin polarization and also pseudo-spin-polarized current are injected in silicene due to the external perpendicular electric field which determines the silicene phase. Applying this field leads to inversion symmetry breaking.

Another intriguing feature in 2D systems is spin-valley locking. Two inequivalent energy extrema in energy dispersion of these materials introduce ‘valley’ degree of freedom which is very promising in the new research field; ‘vallytronics’^13^. The valley polarization is an imbalance in the electron population in two valleys^13^. The systems which exhibit three following features: Preserving P and T symmetries; owning two well-distinguished energy extrema called valleys which are transformed to each other by T symmetry; and having large spin-orbit coupling; are good candidates for injecting valley-dependent spin polarization by an external electric field. Before applying this electric field, system is spin degenerate and also valley degenerate. By applying electric field, which is responsible for P symmetry breaking, large spin-orbit coupling in silicene leads to spin sub-bands splitting as if they were exposed to a magnetic field. Therefore, this effect is called Zeeman-like splitting^13^. As no external magnetic field is applied, the system is T-symmetric. Therefore, the opposite spin polarizations are formed in two valleys. This spin polarization emerged in valleys is electric-field reversible^13^ i.e. changing the sign of electric field, which transforms two valleys to each other, will lead to flip of spin polarization. This effect is called spin-valley locking that arises as a result of the spin-orbit coupling and normal electric field which lifts the band degeneracy at the Dirac points. This provides a framework in which the valley polarization results in spin polarization and vice versa^14^. In spite of lack of knowledge about spin and valley relaxation timescales, it has been reported that spin-valley locking effect enhances spin and valley relaxation times^13,15^. Another interesting feature of this effect is the ability of controlling the valley polarization via lifting the valley degeneracy^13^. In the present study, it has been shown that spin-valley locking provides a powerful framework for magnetic-based valley polarization measurement. In addition, it has been demonstrated that the inversion symmetry breaking normal electric field enhances the light induced pseudo-spin polarization and pseudo-spin current significantly.

## Method

**Figure 1:**
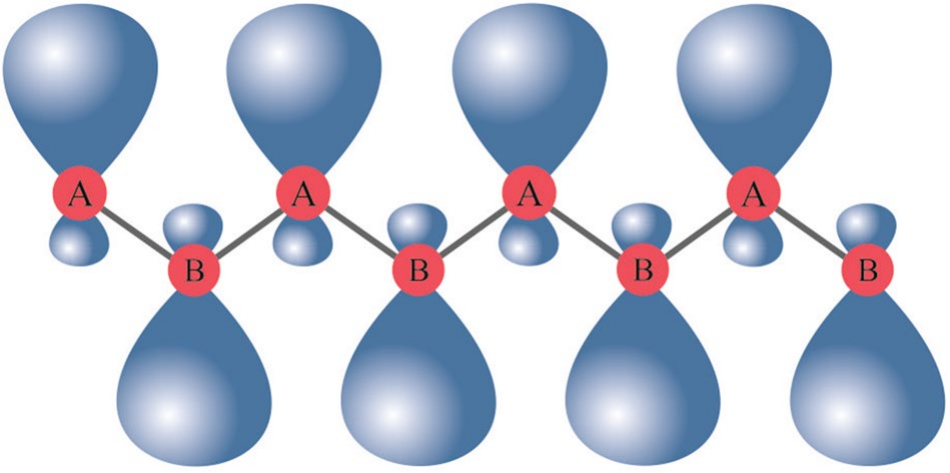
Schematic side view of the silicene $\pi$ orbitals in buckled configuration.

Using $sp^3$ orbitals of *z*-direction (Fig. 1) as $\pi$-bond (see supplementary materials) one can develop the tight-binding approach as described in this section.

More or less analogous to graphene, monolayer graphene-like materials are also honeycomb lattice structures and can be described by the tight-binding model^16,17^. The rather strong intrinsic spin-orbit coupling is responsible for spin dynamics^18^ and makes graphene-like structures topological insulators^5^. In addition, there is a layer separation between the two sublattices in their buckled structure which makes the gap tunable by applying a perpendicular electric field, $E_z$^5^. Therefore the Hamiltonian describing graphene-like materials takes the following form^16,17^. The values of parameters vary for different graphene-like structures. Hamiltonian of the silicene is given by the following expression:

1 $$\begin{aligned} H=H_0+H_{SO}+H_{intR}+H_{extR}+H_b \end{aligned}$$

or

2 $$\begin{aligned} H&=  -t\sum _{\langle {ij}\rangle \alpha } c^{\dagger }_{{i\alpha }}c_{{j\alpha }}+it_{{SO}}\underset{\langle \langle {ij}\rangle \rangle \alpha \beta }{\sum u_{ij}}c^{\dagger }_{{i\alpha }}\left( \sigma ^z\right) _{\alpha \beta }c_{{j\beta }}-{it}_{{intR}}\underset{\langle \langle {ij}\rangle \rangle \alpha \beta }{\sum \mu _{\mathrm{ij}}}c^{\dagger }_{{i\alpha }}\left( \overset{\rightharpoonup }{\sigma }\times \overset{\rightharpoonup }{d}_{{ij}}\right) ^z_{\alpha \beta }c_{{j\beta }}\nonumber \\&\quad + it_{{extR}}\sum _{\langle {ij}\rangle \alpha \beta } c^{\dagger }_{{i\alpha }}\left( \overset{\rightharpoonup }{\sigma }\times \overset{\rightharpoonup }{d}_{{ij}}\right) ^z_{\alpha \beta }c_{{j\beta }}+l\sum _{i\alpha } \zeta _iE_z^ic^{\dagger }_{{i\alpha }}c_{{i\alpha }} \end{aligned}$$

where $$c_{i\alpha }^\dagger $$ creates an electron with spin polarization $$\alpha $$ and orbital state of $$\phi ^{(1)}_{sp^3}$$ at site *i* and $$c_{i\alpha }$$ annihilates an electron with spin polarization $$\alpha $$ and $$\pi $$-orbital state $$\phi ^{(1)}_{sp^3}$$ at site *j* (see supplementary materials). Where, $$<i,j>$$$$(<<i,j>>)$$ run over all the nearest (next nearest) neighbor hopping sites. The first term represents nearest-neighbor hopping in which *t* is the hopping energy. The second term represents the effective spin-orbit coupling where $$\overset{\rightharpoonup }{u}_{ij}=\frac{\vec {d}_i\times \vec {d}_j}{\left| {\vec {d}}_i\times {\vec {d}_j}\right| }$$, $$\vec {d}_i$$ and $$\vec {d}_j$$ denote the nearest bonds that connect the next nearest neighbors and $$\vec {d}_{ij}=\vec {d}_i-\vec {d}_j$$. In addition, $$u_{ij}=1$$ or $$u_{ij}=-1$$ in case that next- nearest neighbor hopping is counterclockwise or clockwise respectively. for the electrons in the sublattice *A*, $$\mu _{ij}=+1$$ and for the electrons in the sublattice *B*, $$\mu _{ij}=-1$$. $$t_{SO}$$ is the strength of the effective spin-orbit coupling and the Pauli matrix in the spin space is denoted by $$\sigma $$. The third term represents the intrinsic Rashba spin-orbit coupling related to the next-nearest-neighbor hoping. The forth term indicates the external Rashba spin-orbit coupling which is associated with the first-nearest-neighbor hoping. As a consequence of inversion symmetry breaking, this term could be induced by an external electric field or a substrate. The last term is the sublattice potential term which arises from structural buckling. *l* stands for the buckling height, $$\zeta _i=+1(-1)$$ for the sublattice *A*(*B*) degree of freedom and $$E_z$$ is the external electric field perpendicular to the 2D sheet which can control the amount of staggered sublattice potential, $$lE_z$$. Based on previous reports, numerical values of these parameters have been given in supplementary materials. Moreover, real-space Hamiltonian can be transformed into the *k*-space using the relation $$c_{i \alpha }=\sum _{i}exp(-i\vec {k}.\vec {R}_i)c_{k\alpha }$$ in which $$\vec {k}=(k_x,k_y)$$ is the wave number of the charged carriers and $$R_i$$ denotes the atomic positions. Then the *k*-space Hamiltonian reads as follow

3 $$\begin{aligned} H=\left( \begin{array}{cccc} \eta +lE_z &{} 0 &{} \gamma _k &{} i \beta _+ \\ 0 &{} -\eta +lE_z &{} i\beta _- &{} \gamma _k \\ \gamma _k^* &{} -i\beta _-^* &{} -\eta -lE_z &{} 0 \\ -i\beta _+^* &{} \gamma _k^* &{} 0 &{} \eta -lE_z \\ \end{array} \right) . \end{aligned}$$

where we have defined following parameters

4 $$\begin{aligned} \eta&=  t_{SO}(2\sin (k_ya)-4\sin \left( \frac{\sqrt{3}}{2}k_xa\right) \cos (\frac{k_ya}{2}))\nonumber \\ \left| \gamma \right| ^2&=  t\left( 1+4\cos \left( \frac{\sqrt{3}}{2}k_ya\right) \cos \left( \frac{3}{2}k_xa\right) +4\cos ^2\left( \frac{\sqrt{3}}{2}k_ya\right) \right) \end{aligned}$$

and also $\beta _\pm =\beta _1\pm \beta _2$ in which

5 $$\begin{aligned} \beta _1= & {} t_{extR}\exp \left( -i\frac{k_xa}{2\sqrt{3}}\right) \sin \left( \frac{k_ya}{2}\right) \end{aligned}$$

6 $$\begin{aligned} \beta _2= & {} \frac{\sqrt{3}}{3}t_{extR}\left( \exp \left( i\frac{k_xa}{\sqrt{3}}\right) +\exp \left( -i\frac{k_xa}{\sqrt{3}}\right) \cos \left( \frac{k_ya}{2}\right) \right) \end{aligned}$$

In which, we have ignored the intrinsic Rashba interaction in the numerical computations to provide a clear understanding of the most important factors in the light induced effects.

The interaction between the electrons and electromagnetic field in length gauge and long-wavelength approximation inside the silicene has the form^19^

7 $$\begin{aligned} V=e\int d\overset{\rightharpoonup }{r}{\hat{\psi }}^{\dagger }(\overset{\rightharpoonup }{ {r}}) (\overset{\rightharpoonup }{r}.\overset{\rightharpoonup }{E}(t) ){\hat{\psi }} (\overset{\rightharpoonup }{r}). \end{aligned}$$

By expanding the field operators in terms of the silicene wave functions

8 $$\begin{aligned} {\hat{\psi }} (\overset{\rightharpoonup }{r})=\frac{1}{\sqrt{N}}\sum _{k,R_A} e^{-i\vec {k}.\vec {R}_A}\phi ^{(1)} _{ {sp}^3}\left( \vec {r}-\vec {R}_A\right) a_k+\sum _{k,R_B} e^{-i\vec {k}.\vec {R}_B}\phi ^{(1)} _{ {sp}^3}\left( \vec {r}-\vec {R}_B\right) b_k, \end{aligned}$$

where $a^\dagger _k$ ($a_k$) and $b^\dagger _k$ ($b_k$) are creation (annihilation) operators which create (annihilate) an electron with wave vector *k* in *A* and *B* sublattices respectively. Using above relations one can write

9 $$\begin{aligned} V&=  \sum _{k,k^{\prime}}(\overset{\rightharpoonup }{D}_{ {AA}}.\overset{\rightharpoonup }{E}(t)a^{\dagger }_{k^{\prime}}a_k+\overset{\rightharpoonup }{D}_{{AB}}.\overset{\rightharpoonup }{E}(t)a^{\dagger }_{k^{\prime}}b_kf(k)+\overset{\rightharpoonup }{D}_{{BA}}.\overset{\rightharpoonup }{E}(t)b^{\dagger }_{k^{\prime}}a_k f^*(k)+\overset{\rightharpoonup }{D}_{{BB}}.\overset{\rightharpoonup }{E}(t)b^{\dagger }_{k^{\prime}}b_k)\delta _{{k,k}^{\prime}}\nonumber \\&=  \left( \begin{array}{cc} \overset{\rightharpoonup }{D}_{\mathrm{AA}}.\overset{\rightharpoonup }{E}(t) &{} \overset{\rightharpoonup }{D}_{\mathrm{AB}}.\overset{\rightharpoonup }{E}(t)f(k) \\ \overset{\rightharpoonup }{D}_{\mathrm{BA}}.\overset{\rightharpoonup }{E}(t)f^*(k) &{} \overset{\rightharpoonup }{D}_{\mathrm{BB}}.\overset{\rightharpoonup }{E}(t) \\ \end{array} \right) \otimes I_S \end{aligned}$$

In which $I_S$ is the identity operator in spin space and electric dipole moment is defined by the following relation

10 $$\begin{aligned} \overset{\rightharpoonup }{D}_{\alpha \beta }=\int d^3r\phi ^{(1)*} _{\mathrm{sp}^3}(\vec {r}-\vec {R}_\alpha )e\overset{\rightharpoonup }{r}\phi ^{(1)} _{\mathrm{sp}^3}\left( \vec {r}-\vec {R}_\beta \right) ,~~(\alpha ,\beta =A,B) \end{aligned}$$

and $f(k)=\sum _i e^{i\overset{\rightharpoonup }{k}.\overset{\rightharpoonup }{\delta }_i}$ where $\overset{\rightharpoonup }{\delta }_i$ are the nearest neighbors position vectors. Electric part of the light emission is identified by $\overset{\rightharpoonup }{E}(t)=E(t){\hat{\epsilon }}_p$ in which ${\hat{\epsilon }}_p$ denotes the light polarization and in the present work has been assumed to be ${\hat{\epsilon }}_p=(1,i,0)$ for a circularly polarized incident wave.

Eventually, the light-matter interaction takes the form

11 $$\begin{aligned} V=\left( \begin{array}{cccc} \overset{\rightharpoonup }{D}_{ {AA}} &{} 0 &{} f(k)\overset{\rightharpoonup }{D}_{ {AB}} &{} 0 \\ 0 &{} \overset{\rightharpoonup }{D}_{ {AA}} &{} 0 &{} f(k)\overset{\rightharpoonup }{D}_{ {AB}} \\ f^*(k)\overset{\rightharpoonup }{D}_{ {BA}} &{} 0 &{} \overset{\rightharpoonup }{D}_{ {BB}} &{} 0 \\ 0 &{} f^*(k)\overset{\rightharpoonup }{D}_{ {BA}} &{} 0 &{} \overset{\rightharpoonup }{D}_{ {BB}} \\ \end{array} \right) \cdot \overset{\rightharpoonup }{E}(t) \end{aligned}$$

where according to Eq. (10), it can be shown that the off-diagonal elements of the dipole moment i.e. $D_{AB}$ and $D_{BA}$ are relatively small ${\overset{\rightharpoonup }{D}_{{AB}}=\overset{\rightharpoonup }{D}_{{BA}}\simeq \left( 5.96\times 10^{-5}, -6.13\times 10^{-10}, -5.12\times 10^{-5}\right) }$ (in the unit of electron-Angstrom; $e\AA$) in comparison with diagonal elements, $D_{AA}$ and $D_{BB}$ that have considerable values given by $\overset{\rightharpoonup }{D}_{{AA}}=\overset{\rightharpoonup }{D}_{{BB}}\simeq \left( 1.915\times 10^{-5}, -4.112\times 10^{-7}, -0.240\right)$ ($e\AA$).

It should be noted that even small off-diagonal elements of the dipole moment have to be considered. Ignoring these components leads to a diagonal *V* matrix which cannot result in inter-band transitions and non-equilibrium polarization injection.

According to Inglot et al.^18^, the injection rate of any quantity ${\hat{O}}$ is obtained using the well-known Fermi’s golden rule,

12 $$\begin{aligned} O(\omega )&= \sum _{n,n^{\prime}} O^{n\rightarrow n^{\prime}}(\omega )\nonumber \\ O^{n\rightarrow n^{\prime}} {(\omega )}&= {\frac{2\pi }{\hbar }} {\int \frac{d^2k}{(2\pi )^2}}\left| \left\langle \psi _{ {nk}}\left| {\hat{V}}\right| \psi _{n^{\prime}k}\right\rangle \right| ^2{\hat{O}}^{n\rightarrow n^{\prime}}\times \delta (E_{ {nk}}+\hbar \omega -E_{n^{\prime}k})f(E_{ {nk}})(1-f(E_{n^{\prime}k})) \end{aligned}$$

where *n* and $n^{\prime}$ are the numbers of sub-bands which optical transitions occur within and $f(E_{ {nk}})$ is the Fermi-Dirac distribution function. Then the non-equilibrium quantities such as spin injection

13 $$\begin{aligned} S_z^{n\rightarrow n^{\prime}}=\left\langle \psi _{n^{\prime}k}\left| \hat{s_z}\right| \psi _{n^{\prime}k}\right\rangle -\left\langle \psi _{ {nk}}\left| \hat{s_z}\right| \psi _{ {nk}}\right\rangle , \end{aligned}$$

non-equilibrium normal pseudo-spin

14 $$\begin{aligned} \tau _z^{n\rightarrow n^{\prime}}=\left\langle \psi _{n^{\prime}k}\left| \hat{\tau _z}\right| \psi _{n^{\prime}k}\right\rangle -\left\langle \psi _{ {nk}}\left| \hat{\tau _z}\right| \psi _{ {nk}}\right\rangle , \end{aligned}$$

and pseudo-spin current

15 $$\begin{aligned} J_{\tau _z}^{ n\rightarrow n^{\prime}}=\left\langle \psi _{n^{\prime}k}\left| {\hat{J}}^{\tau _z}\right| \psi _{n^{\prime}k}\right\rangle -\left\langle \psi _{ {nk}}\left| {\hat{J}}^{\tau _z}\right| \psi _{ {nk}}\right\rangle \end{aligned}$$

could be defined within this approach where ${\hat{J}}^{\tau _z}_{\alpha }= \frac{1}{2}({\hat{v}}_\alpha {\hat{\tau }}_z+{\hat{\tau }}_z {\hat{v}}_\alpha )$ and ${\hat{v}}_\alpha = \frac{1}{\hbar }\frac{\partial {\hat{H}}}{\partial k_\alpha }$ is the band velocity along the $\alpha$-direction.

$O^{n\rightarrow n^{\prime}}$ measures the change of expectation value of *O* via the transition from initial energy band *n* to band $n^{\prime}$ i.e. $O^{ n\rightarrow n^{\prime}}=\left\langle \psi _{n^{\prime}k}\left| {\hat{O}}\right| \psi _{n^{\prime}k}\right\rangle -\left\langle \psi _{ {nk}}\left| {\hat{O}}\right| \psi _{ {nk}}\right\rangle$ . Therefore, induced polarization represents the variation of operator *O* due to transitions i.e. the non- equilibrium polarization of system due to optical pumping.

In the light absorption process, at the range of photon energies that are slightly greater than the band gap, there is another key factor which specifies how much a transition could be effective in generation of polarization. The electron-hole asymmetry is a consequence the Rashba spin-orbit interaction^20^. For those transitions that take place between valence and conduction bands particle-hole asymmetry can enhance the non-equilibrium values of a given observable. Asymmetry between the conduction and valence bands results in more effective transitions in which the change of expectation values for excited electron is high.

## Results and discussion

Numerical results which have been represented here are normalized to electrons density of the system, $n_0\simeq 1.5\times 10^{15}cm^{-2}$ and temperature given in the distribution functions has been chosen to be $T=1$K.

### The influence of staggered potential

In the case of graphene, applying a large external magnetic field is crucial for observing photogalvanic effect and light induced spin polarization^18^. In this case Fermi surface deformation caused by external magnetic field, has been considered to be responsible for non-equilibrium spin-current^18^. However, in the case of silicene, significant photon induced polarizations can be injected even at zero magnetic field but in the presence of a normal static electric field. The spin-orbit coupling in silicene is strong enough and can sufficiently lead to band energy splitting, meanwhile, normal electric field results in pseudo-spin polarized bands and spin polarized valleys close to the Dirac points. When the perpendicular static electric field is applied to silicene sheet, due to different responses of silicon atoms at each of the sublattices, there exists a potential difference between two sublattices which is known as *staggered potential*^21^. Exactly analogous to an external magnetic field which can induce a net spin polarization, this static perpendicular electric field can induce pseudo-spin polarization. In this work, the role of perpendicular static electric field in light induced polarizations has been numerically studied. It has been observed that in the absence of this field, there is no non-equilibrium pseudo-spin polarization. By switching on the normal electric field, significant non-equilibrium polarization can be generated. In addition, external electric field remarkably enhances other light induced polarizations such as pseudo-spin current.

**Figure 2:**
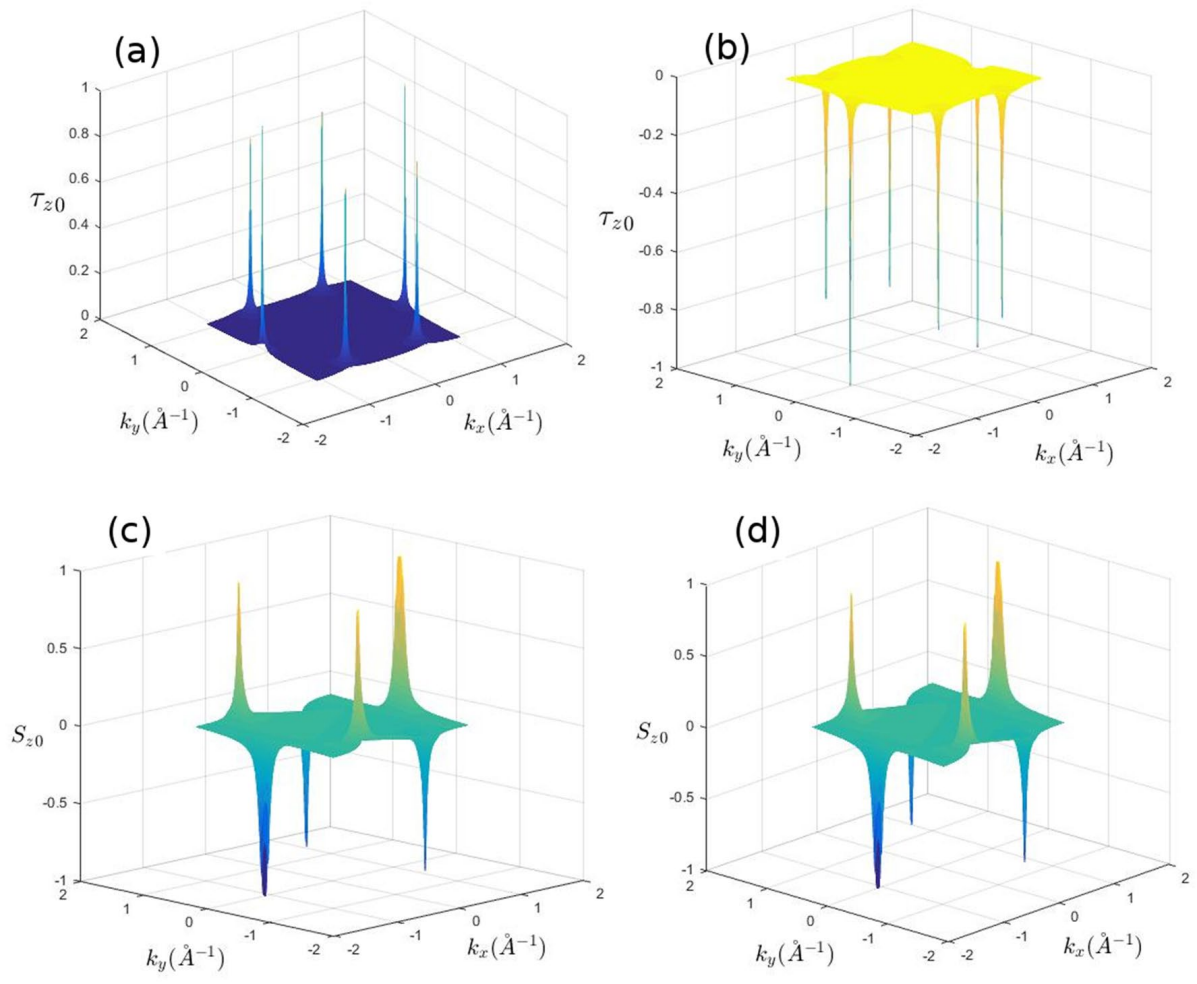
Equilibrium spin and pseudo-spin polarizations in silicene. (**a**) Pseudo-spin polarization of the first conduction band (n = 3). (**b**) Pseudo-spin polarization of the second valance band (n = 2). (**c**) Spin polarization of the second valance band (n = 2). (**d**) Spin polarization of the first conduction band (n = 3). States are fully spin and pseudo-spin polarized very close to the Dirac points and become partially polarized away from these points.

Applying the perpendicular static electric field, which produces a staggered potential between two sublattices, breaks the inversion symmetry and subsequently induces a net pseudo-spin polarization at equilibrium (Fig. 2a,b) around the Dirac points. As shown in the Fig. 2a,b each band has a specified normal pseudo-spin polarization at Dirac points where the successive band has opposite pseudo-spin polarization. However, as it has been shown in this work pseudo-spin polarized bands provide more effective framework for light induced polarizations. Normal electric field is responsible for pseudo-spin polarized bands at equilibrium. Meanwhile, it should be noted that the non-equilibrium light induced pseudo-spin polarization has also been enhanced remarkably by the staggered filed.

The main role of the perpendicular electric field can be clearly seen in equilibrium condition i.e. in the absence of radiation field when silicene sheet is exposed to perpendicular Electric field, $E_z$, there will exist a net pseudo-spin equilibrium polarization.

As it can be seen in the Fig. 2a,b, in the absence of light emission, applying perpendicular electric field solely can induce a net pseudo-spin polarization in both valence and conduction bands. Normal component of pseudo-spin, $\tau _z$, is a good quantum number close to the Dirac points. Therefore, it is noticeable that any factor which causes an inter-band transition, this band change is also accompanied with a pseudo-spin flip of electron.

On the other hand, as mentioned earlier, normal electric field results in spin-valley locking which is almost band-independent. This can be inferred from the Fig. 2c,d. This normal spin preserving property of electrons at each of the valleys has been removed by lifting the normal electric field.

The pseudo-spin polarized bands and spin-valley locking which has been observed at the vicinity states of the Dirac points can be explained simply within the Dirac point approximation. Given that extrinsic Rashba coupling is small (that is about 0.001eV), in the presence of perpendicular electric field and very close to the Dirac points ($k\rightarrow 0$), low energy Dirac Hamiltonian $H^{\eta }_D = \hbar v_F\left( \eta k_x \tau _x+k_y \tau _y\right) + \eta \tau _z \left( a \lambda _{ R2}\left( k_y \sigma _x-k_x \sigma _y\right) \right) +\eta \lambda _{SO} \tau _z\sigma _z -l E_z \tau _z$ is reduced as $H = diag\{\eta \lambda _{ {SO}}+ {lE}_z,-\eta \lambda _{ {SO}}+ {lE}_z, -\eta \lambda _{ {SO}}- {lE}_z, \eta \lambda _{ {SO}}- {lE}_z \}$ in which $\lambda _{ {SO}}$ and $\lambda _{ R2}$ are spin-orbit coupling constant and intrinsic Rashba interaction strength respectively. Accordingly, in this case the Hamiltonian is diagonal in the bases of $|\tau _z>\otimes |\sigma _z>$ which means that the eigenstates have definite pseudo-spin and spin quantum numbers. Eigenvalue of this eigenstate is given by $\varepsilon ^\eta _{\tau \sigma }= \eta \sigma _z \lambda _{ {SO}}+ \tau _z{lE}_z$ where $\varepsilon ^\eta _{\tau \sigma }=\varepsilon ^{-\eta }_{\tau -\sigma }$ clearly exhibits spin-valley locking that electrons of the same band and different valleys are isoenergetic when their spins directed oppositely. Meanwhile, there is a gap between the pseudo-spin up and pseudo-spin down states given by $\Delta =2|lE_z-\lambda _{SO}|$ which indicates that at zero electric field ($E_z=0$) the energy difference between up and down spins is very small of order of spin-orbit coupling and therefore population difference between the opposite spins is negligible. These results directly reflect the time-reversal symmetry of the system when the inversion symmetry is broken. In addition, this electrically tunable band gap provides a topological phase transition by manipulation of the normal electric field^5,16^.

### Light-induced spin and valley polarization

In the presence of staggered potential and due to spin-valley locking, spin population imbalance is almost identical with the valley polarization at low Fermi energies. In other words, since non-equilibrium spin polarization coincides with the population imbalance between the two valleys, spin polarization measures indirectly the valley polarization.

At first look it seems that the spin-valley locking results in exactly identical population of $s_z$-spins with its corresponding valley population. However, it should be noted that all of the occupied states in a single valley are not completely $s_z$ polarized. Equilibrium spin polarization of a given state depends on the position of this state in *k*-space. Very close to the Dirac points states are fully spin polarized and as the distance of the state from the Dirac point increases, spin polarization of the state decreases (see supplementary materials). At low Fermi energies, the spin and valley populations are very close while away from the Dirac points states are partially polarized. Assuming that the up (down) spins are located around the *K* ($K^{\prime}$) valley (as a result of the spin-valley locking) the relation between the spin and valley population at each of the valleys can be given by the following expressions, $n_\uparrow = \alpha _K n_K$ and $n_\downarrow = \alpha _{K^{\prime}} n_{K^{\prime}}$. Where, $n_K$ ($n_{K^{\prime}}$) stands for the valley population of *K* ($K^{\prime}$) valley and $n_\uparrow$ ($n_\downarrow$) is the spin population of the same valley. $\alpha _\eta$ is a coefficient that measures the ratio of the polarized states in the $\eta$-valley which depends on the Fermi wave number ($k_F$). Due to the symmetry of the valleys one can expect that $\alpha _{K}=\alpha _{K^{\prime}} =\alpha$ and accordingly one can easily obtain $P_s = \alpha ~ P_v$ where $P_s=(n_\uparrow -n_\downarrow )/n$ and $P_v=(n_K-n_{K^{\prime}})/n$ are the spin polarization and valley polarization of the system respectively. Meanwhile, the $n =n_K+n_{K^{\prime}}$ is the electronic population of the sample. $\alpha$ can simply be estimated by the following relation,

16 $$\begin{aligned} \alpha _K=\frac{1}{(\hbar /2) n}<Sz>_K, \end{aligned}$$

in which $<Sz>_K$ is sum of the expectation value of ${\hat{s}}_z$ over all of the occupied states in a single valley. Therefore, spin and valley polarization can be related to each other via $P_s = \alpha ~ P_v$.

Behavior of the system depends on the chirality of circularly polarized incident light. When the silicene sheet is exposed to circular right-handed polarized light, the behaviour of spin in *K* valley is exactly the same as $K^{\prime}$ valley in the presence of left-handed light and vice-versa. Accordingly, it is expected that each of these circularly polarized lights could provide one of the valley polarizations in the silicene^14^.

**Figure 3:**
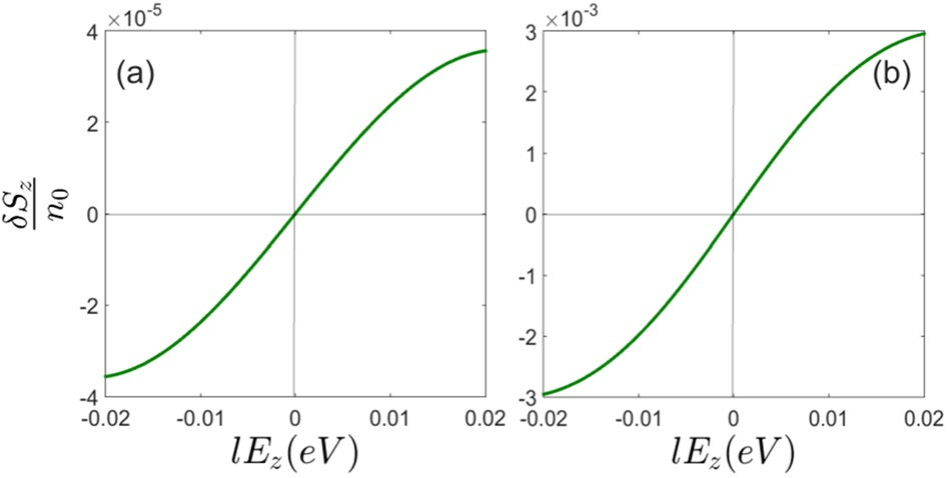
Spin polarization of (**a**) *K* and (**b**) $K^{\prime}$ valleys in terms of the normal electric field at $\hbar \omega =0.3eV$.

As indicated in Fig. 3 spin polarization is suppressed at $E_z = 0$. This can be explained by the concept of the spin-valley locking and non-symmetric light absorption of the left and right-handed photons. As discussed earlier, light induced spin polarization in silicene can be considered as a direct consequence of the spin-valley locking that has been induced by the normal external electric field.

### Detection of valley-polarization

**Figure 4:**
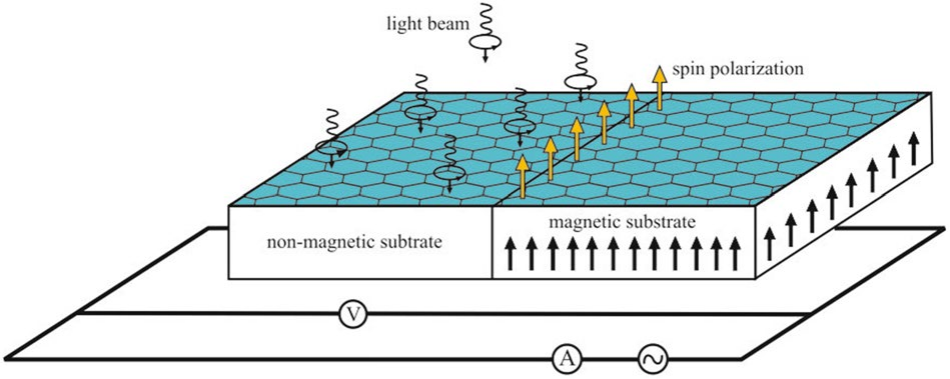
Spin based detection of the valley polarization in which the light induced valley polarization could be measured by its corresponding spin polarized population. The spin polarization is measured by a simple magneto-resistance setup.

Large separation between two valleys in momentum space, leads to a long inter-valley scattering time. This can grant the valley degree of freedom an opportunity to perform the role of information carrier in valleytronics^22^. Since, the valley population imbalance is proportional to spin polarization by the spin-valley locking effect, it seems that the spin polarization detection devices could be proposed for valley polarization measurements as well. Therefore, a simple magneto-resistance detection setup can be employed. As shown in the Fig. 4 this setup includes silicene sheet deposited on a bipartite substrate. One half is non-magnetic and the other half is magnetic. As depicted in this figure an ac current can provide light modulation which could be utilized at low frequencies.

By applying the electromagnetic radiation on non-magnetic part, if the light-induced magnetic polarization is aligned with magnetization of ferromagnetic substrate, the voltage in circuit will be changed and magneto-resistance of the sample scales with the spin population imbalance. Then the valley polarization could be obtained via the correspondence of spin and valley population given the relations discussed above.

As discussed in supplementary materials which has also been reported in previous works^13,15^ spin-valley locking enhances spin life-time. Therefore, in the spin-valley locking regime spin relaxation time could be high enough for effective detection of spin polarization in the magnetic region.

### Non-equilibrium pseudo-spin polarization and pseudo-spin current

**Figure 5:**
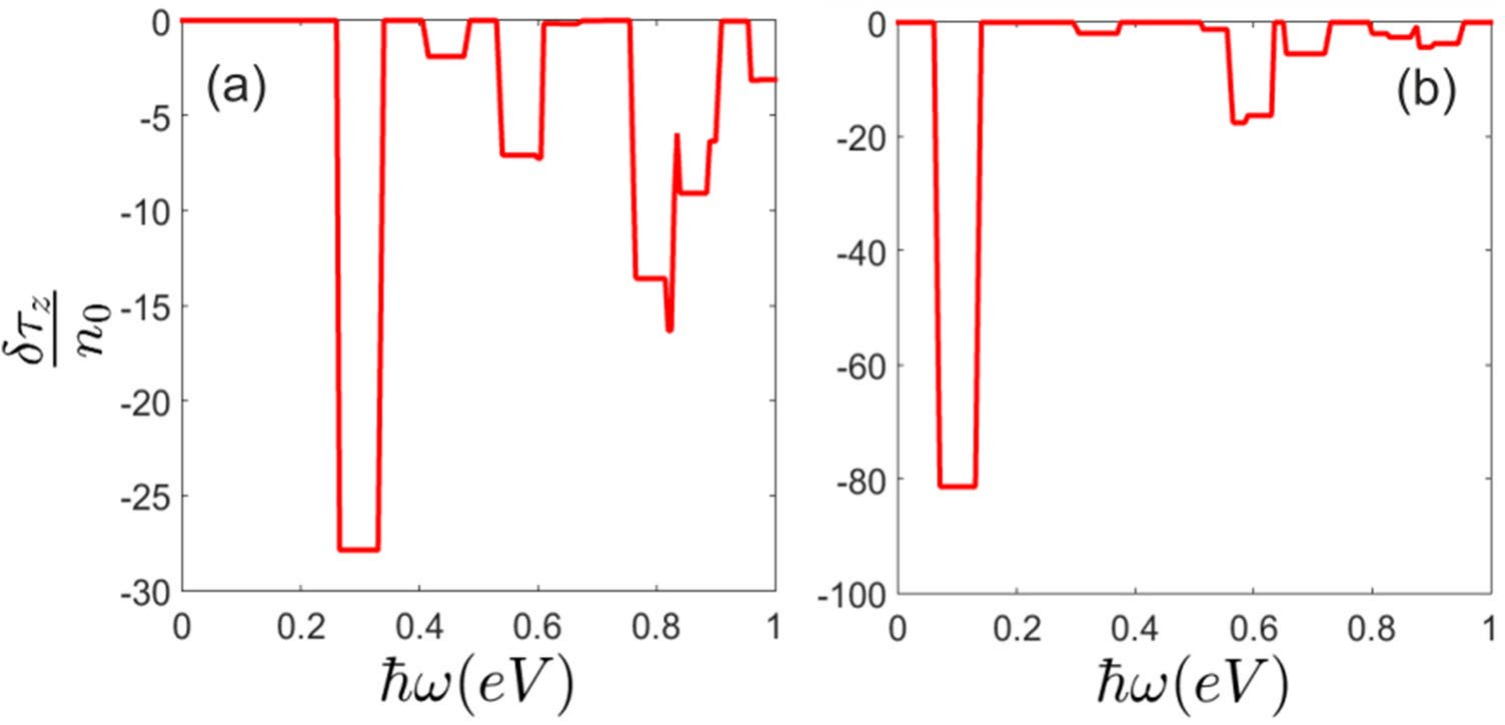
Pseudo-spin polarization of (**a**) *K* and (**b**) $K^{\prime}$ valleys at $lE_z=0.03eV$.

**Figure 6:**
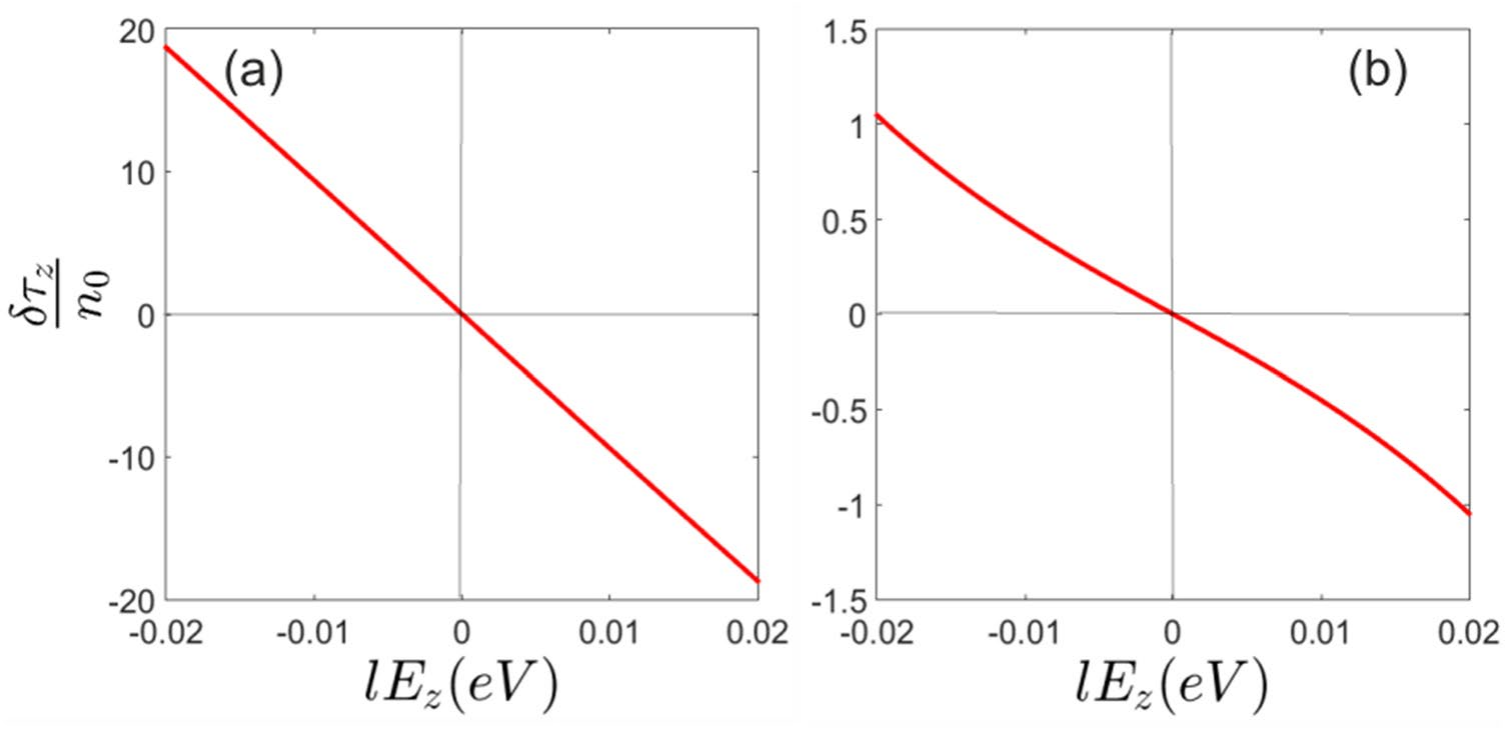
Pseudo-spin polarization of (**a**) *K* and (**b**) $K^{\prime}$ valleys in terms of the normal electric field at $\hbar \omega =0.3eV$.

As it can be inferred from Figs. 5 and 6 non-equilibrium pseudo-spin polarization can be induced by circularly polarized photons. The light-matter interaction leads to a considerable non-equilibrium pseudo-spin polarization when the normal static filed is applied. It should be noted that in the absence of perpendicular electric field, non-equilibrium pseudo-spin polarization cannot be injected by the radiation field (Fig. 6). At zero electric field and very close to the Dirac points band energies are given by $\varepsilon ^\eta _{\tau \sigma }= \eta \sigma _z \lambda _{ {SO}}$ where the eigenstates are $|\tau _z>\otimes |\sigma _z>$ and therefore pseudo-spin up and down states located near to these points become degenerate at $E_z =0$. This gives rise to zero pseudo-spin polarization. On the other hand, states that have been placed far from the Dirac points are not pseudo-spin polarized and light induced transitions between these states could not result in effective non-equilibrium pseudo-spin polarization.

Meanwhile, since successive pseudo-spin dependent bands in the presence of normal field have opposite sign of pseudo-spin polarization, light induced transitions between these bands result in negative non-equilibrium pseudo-spin polarization (Fig. 6).

**Figure 7:**
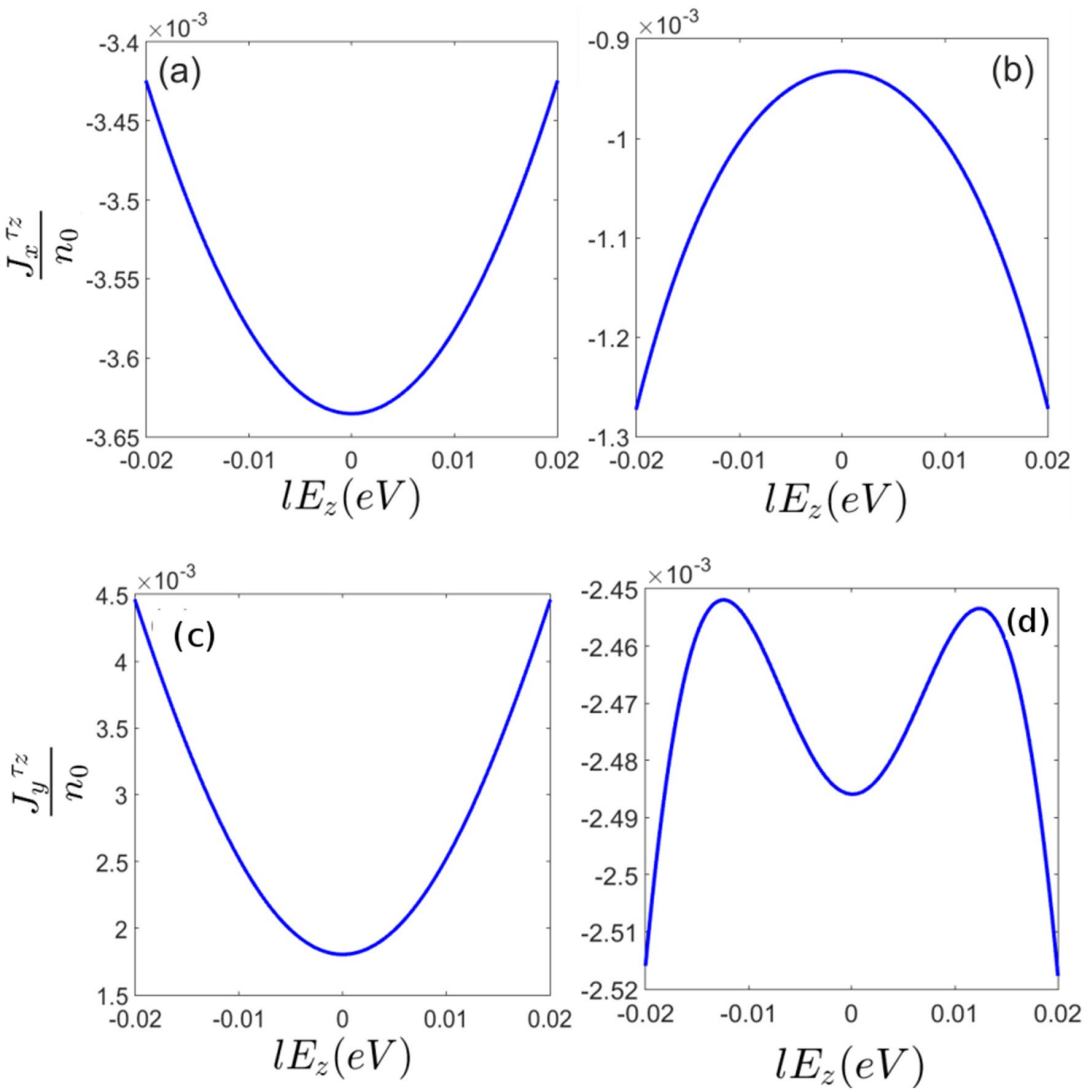
Light induced pseudo-spin current of (**a**) *K* and (**b**) $K^{\prime}$ valleys along the *x*-axis in terms of the normal electric field at $\hbar \omega =0.3eV$. Similarly, light induced pseudo-spin current of (**c**) *K* and (**d**) $K^{\prime}$ valleys along the *y*-axis in terms of the normal electric field at $\hbar \omega =0.3eV$.

Light induced transitions can result in non-equilibrium pseudo-spin current in silicene. Figure 7a–d represent pseudo-spin current along the *x* and *y* axis respectively. It can be inferred that pseudo-spin-polarized currents are different in two perpendicular directions *x* and *y*. This means that optical radiation not only injects valley polarization in silicene sheet but also results in anisotropic response of the system. This anisotropy would rely on the silicene band anisotropy which can be captured beyond the Dirac point approximation as performed in the present study. Within this approach band anisotropy manifests itself in the numerical results.

Since the circularly polarized photon absorption is not symmetric at different valleys, the population imbalance between the *K* and $K^{\prime}=-K$ results in electric current. Therefore pseudo-spin polarized current increases by normal electric field based on the background pseudo-spin polarization increment.

**Figure 8:**
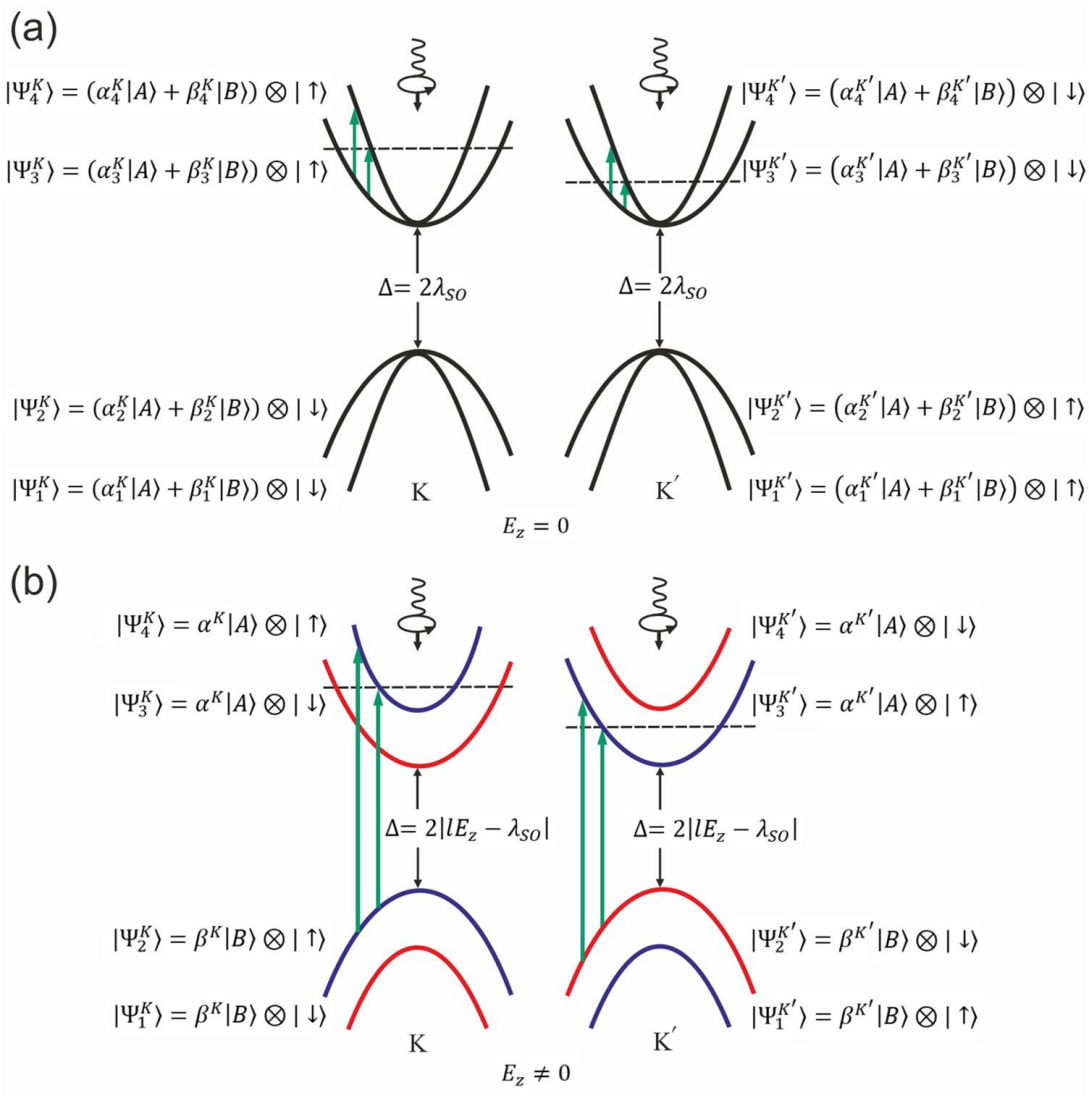
Band energies and light absorption of different valleys in the presence and absence of the normal electric field. Vertical green arrows show the spin preserving light induced transitions. Horizontal dashed lines show photoexcited occupation level. (**a**) Black curves indicate unpolarized pseudo-spin bands at $E_z=0$. (**b**) Blue and red curves indicate the spin and pseudo-spin polarized bands at $E_z\ne 0$. Differently occupied valleys ($K^{\prime}=-K$) at both $E_z=0$ and $E_z\ne 0$ fields and also non-symmetric photon absorption around each of valleys provide pseudo-spin current. Meanwhile, transitions between the degenerate and nearly degenerate states around the Dirac points at $E_z=0$, could not generate accountable spin or pseudo-spin non-equilibrium polarization. On the other hand when $E_z\ne 0$ transitions between the opposite pseudo-spin resolved bands results in major change of average pseudo-spin.

However, it is really remarkable that unlike the non-equilibrium spin and pseudo-spin polarizations, pseudo-spin current could be generated even at zero normal electric field (Fig. 7a–d). This reflects the fact that not only the light absorption of different valleys are not symmetric but also this absorption is not identical for each of the states around a single Dirac point. On the other words, in the absence of the staggered potential each of the band states has different pseudo-spin polarization, $<\tau _z>$, depending on the location of the state in *k*-space and band number, however, the total pseudo-spin polarization of the band is zero. When the circularly polarized light excites non-symmetrical states around the Dirac point overall induced pseudo-spin current becomes nonzero Fig. 8a,b.

Meanwhile, this can be considered as a specific consequence of the present approach which goes beyond the Dirac point approximation. Within the Dirac point approximation pseudo-spin current operator is given by $J^{\tau _z}_{x(y)}=v_F\frac{1}{2}(\tau _z\tau _{x(y)}+\tau _{x(y)}\tau _z)$ (when the internal Rashba interaction has been ignored) that identically vanishes which shows that non-equilibrium pseudo-spin current cannot be captured within the Dirac point approximation.

## Concluding remarks

Within the semi-classical approach light-induced polarization in silicene has been investigated beyond the Dirac point approximation. Calculations are performed at the long-wavelength limit wherein the amplitude of external field remains practically constant over the atomic scale, where light-matter interaction at this limit cannot lead to inter-valley transition.

It has been shown that the normal electric field results in light induced spin and pseudo-spin polarization. Meanwhile, this field enhances the light induced pseudo-spin current.

Non-symmetric absorption of circularly polarized light are known to be responsible for spin polarization and pseudo-spin current. Meanwhile, it is noteworthy, since inside each valley initial and final states of transition possess different pseudo-spins. Therefore, light induced transitions from one energy band to another leads to pseudo-spin flip i.e. negative non-equilibrium pseudo-spin polarization. Spin-valley locking provides a magnetic framework for detection of the valley polarization by non-equilibrium spin measurements.

## Supplementary information

Supplementary Information.
