# Supplementary material for: Normal electric field enhanced light-induced polarizations and magnetic detection of valley polarization in silicene
Source: Sci Rep. 2020 Oct 6;10:16612. doi: 10.1038/s41598-020-73138-5 (PMC7538594; doi:10.1038/s41598-020-73138-5)
Supplement: Supplementary file 1 — Supplementary Information. [file 41598_2020_73138_MOESM1_ESM.pdf]

Supplementary Information
Normal electric field enhanced light-induced polarizations and magnetic detection of valley polarization in silicene

N. Shahabi* and Arash Phirouznia
Department of Physics, Azarbaijan Shahid Madani University, 53714-161, Tabriz, Iran
Condensed Matter Computational Research Lab. Azarbaijan Shahid Madani University, 53714-161, Tabriz, Iran

I. SYSTEM CHARACTERISTICS

Interesting effects arise as results of buckling in silicene therefore particular interest of the present research is focused on this material [1, 2]. Among different proposed hybridizations, buckled silicene, which would be named as Si(111), is described by the hybridization in the second-nearest neighbor (2NN) tight-binding model. This hybridization is the most desired one due to stability reasons [3–6]. Orbitals form bonds with their second nearest neighbors (2NNs). These bonds are responsible for conduction of the buckled silicene (Si (111)).

In order to get π-bonds of sp^3 orbitals along the z-axis we choose a linear combination of s, p_x , p_y and p_z in which sp^3 orbitals take the forms below:

$$\begin{aligned}
\phi^{(1)}_{sp^3}(\theta,\varphi) &= \frac{1}{2}s(\theta,\varphi)+\frac{\sqrt{3}}{2}p_z(\theta,\varphi) \\
\phi^{(2)}_{sp^3}(\theta,\varphi) &= \frac{1}{2}s(\theta,\varphi)-\frac{1}{\sqrt{12}}p_z(\theta,\varphi)+\sqrt{\frac{2}{3}}p_x(\theta,\varphi) \\
\phi^{(3)}_{sp^3}(\theta,\varphi) &= \frac{1}{2}s(\theta,\varphi)-\frac{1}{\sqrt{12}}p_z(\theta,\varphi)-\frac{1}{\sqrt{6}}p_x(\theta,\varphi)+\frac{1}{\sqrt{2}}p_y(\theta,\varphi) \\
\phi^{(4)}_{sp^3}(\theta,\varphi) &= \frac{1}{2}s(\theta,\varphi)-\frac{1}{\sqrt{12}}p_z(\theta,\varphi)-\frac{1}{\sqrt{6}}p_x(\theta,\varphi)-\frac{1}{\sqrt{2}}p_y(\theta,\varphi)
\end{aligned} \tag{1.1}$$

Where s and p orbitals can be written as

$$\begin{aligned}
s(\theta,\varphi) &= Y_{00}(\theta,\varphi) \\
p_x(\theta,\varphi) &= \frac{1}{\sqrt{2}}\left\{Y_{1,1}(\theta,\varphi)+Y_{1,-1}(\theta,\varphi)\right\} \\
p_y(\theta,\varphi) &= \frac{1}{\sqrt{2}}\left\{Y_{1,1}(\theta,\varphi)-Y_{1,-1}(\theta,\varphi)\right\} \\
p_z(\theta,\varphi) &= Y_{10}(\theta,\varphi)
\end{aligned} \tag{1.2}$$

spherical harmonics read as

$$\begin{aligned}
Y_{00}(\theta,\varphi) &= \frac{1}{\sqrt{4\pi}} \\
Y_{10}(\theta,\varphi) &= \sqrt{\frac{3}{4\pi}}\cos\theta \\
Y_{1,\pm1}(\theta,\varphi) &= \sqrt{\frac{3}{8\pi}}\sin\theta e^{\pm i\varphi}.
\end{aligned} \tag{1.3}$$

*Electronic address: shahabi@azaruniv.ac.ir

TABLE I: Lattice constant and energy scales for graphene and other buckled honeycomb materials [7, 8].

material	a(Å)	t(eV)	t_{SO}(meV)	t_{intR}(meV)	l(Å)
silicene	3.86	1.6	0.75	0.46	0.23
graphene	2.46	2.8	0.00114	—	0

Meanwhile, following radial wave functions have also been employed for defining the hybrid orbitals:

$$\begin{aligned} R_{30}(r) &= \frac{2}{\sqrt{27}}\left(\frac{a}{Z}\right)^{\frac{-3}{2}}\left(1-\frac{2}{3}\left(\frac{Zr}{a}\right)+\frac{2}{27}\left(\frac{Zr}{a}\right)^2\right)e^{-\left(\frac{Zr}{3a}\right)} \\ R_{31}(r) &= \frac{8}{27\sqrt{6}}\left(\frac{a}{Z}\right)^{\frac{-3}{2}}\left(1-\frac{1}{6}\left(\frac{Zr}{a}\right)\right)\left(\frac{Zr}{a}\right)e^{-\left(\frac{Zr}{3a}\right)} \end{aligned} \tag{1.4}$$

$\phi^{(1)}_{sp^3}$ contributes in π-bonds while $\phi^{(2)}_{sp^3}$, $\phi^{(2)}_{sp^3}$ and $\phi^{(4)}_{sp^3}$ contribute in the σ-bonds. Considering only the orbital which form π-bonds, one can write

$$\phi^{(1)}_{sp^3}(r,\theta,\varphi) = \frac{1}{2}R_{30}(r)Y_{00}(\theta,\varphi)+\frac{\sqrt{3}}{2}R_{31}(r)Y_{10}(\theta,\varphi), \tag{1.5}$$

in which $Y_{lm}(\theta,\phi)$ and $R_{nl}(r)$ are the spherical harmonics and atomic radial wave functions respectively.

Overlap of parallel orbitals on adjacent Si atoms which are normal to the structure plane form π bonds in silicene. $\phi^{(1)}_{sp^3}$ orbitals of π-bonds are z-oriented and have the key role in electron and spin transport. The other sp^3 orbitals take part in σ-bonds which are covalent-types bonds and have no direct influence on charge transport.

Parameters of the tight-binding Hamiltonian has been given in Table I where a and t are lattice constant and hopping amplitude respectively. t_{SO} stands for spin-orbit coupling strength and t_{intR} is the internal Rashba coupling strength.

II. SPIN-VALLEY LOCKING

As it is shown in the Fig. 1 in the absence of irradiation there is no net spin polarization. After applying the circularly polarized electromagnetic field, as the charge carriers in two different valleys, K and K', response differently to this specific chirality of light, there will emerge different population of electrons in each of valleys which is known as valley polarization (Fig.1). As a result of the spin-valley locking this valley polarization is proportional to the spin polarization as described in this study. As it is obviously shown up to here, perpendicular electric in silicene represents rich physics from topological phase transition to pseudo-spin band polarization and spin-valley locking [10–12].

III. VALLEY POLARIZATION: GENERATION AND DETECTION

Valley polarization can be generated in different ways in graphene-like materials. Magnetic barrier could be inserted in a strained graphene sheet as a valley filter. By adjusting the strength of strain and including a scalar electric potential, amplitude of valley polarization can be tuned meanwhile, its polarity is controllable by switching the direction of the local magnetic field [13].

Since one can associate an intrinsic magnetic moment to valley index, it is possible to measure this degree of freedom directly. This valley-contrasting magnetic moment is called orbital magnetic moment and originates from self-rotation of Bloch wave packets. Thus coupling between orbital magnetic moment and an external magnetic field can produce valley polarization [14].

There have been introduced several detection methods of valley polarization. Di Xiao et al have measured this quantity as a signal of orbital magnetization. Due to a quantized valley-dependent Hall conductance, applying an external in-plane electric

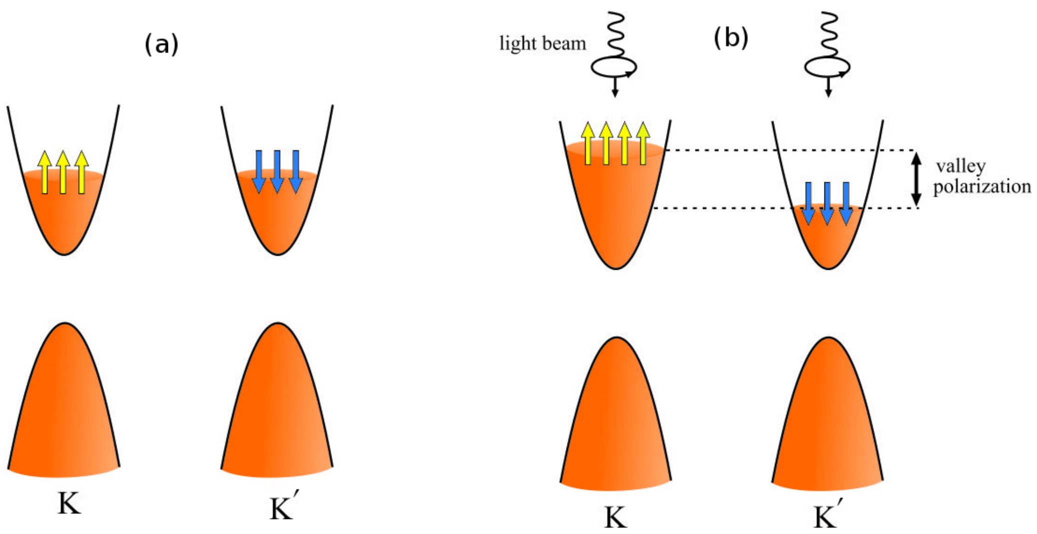

FIG. 1: (a) Normal field induced spin-valley locking. (b) Non-symmetric absorption of the circularly polarized light [9] that generates spin and valley polarization.

field induces a net Hall current which in turn results in a transverse measurable voltage. Therefore the measurement of this voltage can be interpreted as detection of valley polarization [14]. Moreover, it has been shown that by utilizing a universal connection among optical oscillator strength of inter-band transitions, the orbital magnetic moment and the Berry curvature, one can optically measure the orbital magnetization and intrinsic anomalous Hall conductivity in ferromagnetic systems [15]. The breaking of inversion symmetry results in opposite Berry curvatures in two valleys. In the absence of external magnetic field, the net Hall current does not exist but applying a circularly polarized light induces additional populations of electron and holes which their travel to opposite edges of sample generates a transverse voltage. The sign of this voltage indicates the polarization of incident light [15].

There is another method that has been suggested for measurement of valley-polarized current based on transport features in a T-junction of two ribbons which was reported in graphene systems [16]. The measured currents in the armchair and zigzag leads are used to deduce the valley polarization of incoming current [16].

In the present study it has been shown that how spin-valley locking provides a simple magnetic method for detection of the valley polarization. This is originally based on the fact that non-equilibrium spin population and valley population imbalance are proportional to each other as a result of the spin-valley locking.

IV. SPIN AND VALLEY POLARIZATION LIFE TIMES

Since momentum transfer of photon induced transitions is small, this type of state-change cannot be responsible for inter-valley transitions. The momentum difference between two valleys is of the order of $\Delta k \sim |K - K'|$ which is quite large amount in the momentum space for photon induced transitions at energy range where the light absorption rate is not zero [14]. Meanwhile some short-range impurities can provide such a large momentum transfer. It should be reminded that the calculations have been performed beyond the Dirac point approximation where any possible inter-valley transition can be captured within this method.

In the regime of elastic scatterings of impurities, for insulating silicene there is no inter-band induced relaxation thus electron population at each of the valleys remains unchanged. Different type of transitions has been illustrated schematically in Fig. 2. Inter-valley transitions which could be originated from the short range impurities can provide the inter-valley momentum transfers. In addition this type of transitions take place when the impurity potential provides a spin flip of electrons since the different valleys are oppositely spin polarized as a result of spin-valley locking. Therefore an inter-valley transition should be followed by a flip of normal spin component. Therefore, it can be inferred that inter-valley transitions in staggered potential regime just could be provided by sharp (short range) and magnetic impurities.

Spin and valley relaxation times (τ_s and τ_v) are given by $\tau_s \simeq \tau_v \sim \frac{1}{\sum_{K'\lambda'} W^{\xi\xi'}_{\lambda\lambda'}(k,k')}$. Where $W^{\xi\xi'}_{\lambda\lambda'}(k, k')$ is the transition

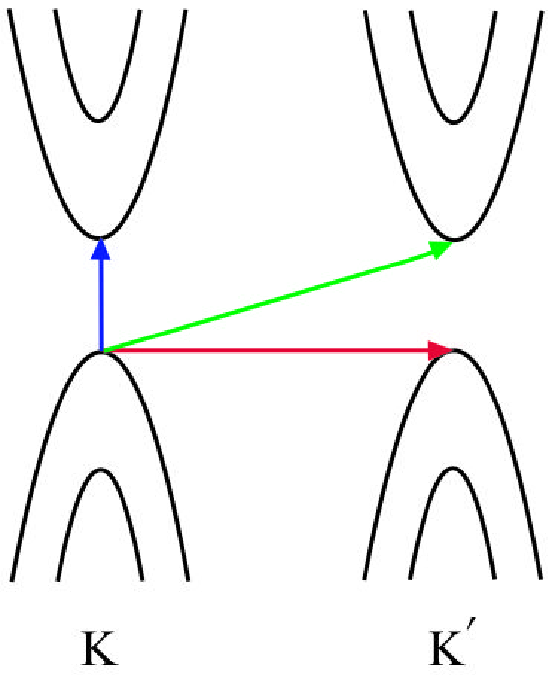

FIG. 2: Blue arrow indicates intra-valley inter-band transition. Red arrow represents intra-band inter-valley elastic transition and green arrow is a schematic example of inter-valley inter-band transition.

rate from $|k\lambda\rangle$ state of ζ valley to $|k'\lambda'\rangle$ of ζ'. At the elastic regime with nonmagnetic impurities one can realize that, $\frac{1}{\tau_s} \simeq \frac{1}{\tau_v} \sim \frac{2\pi v_0^2}{\hbar E_f} n\overline{|\langle u^{\dagger}_{K\lambda}|u_{K'\lambda}\rangle|}^2$. Regardless of the chosen form of electron interaction with light and impurity, in the elastic regime of relaxations, if the short-range impurities are nonmagnetic, the probability of inter-valley transitions for the states in vicinity of Dirac points is practically negligible $< u^{\dagger}_{K\lambda} u_{K'\lambda} > \to 0$ where K and K' are valley indices and λ stands for energy band (band index should not change in the elastic scatterings therefore both states $u_{K\lambda}$ and $u_{K'\lambda}$ have the same band number). Thus the electron states could be considered as *long- live spin-valley* states i.e. spin and valley lifetimes are of the same order and large enough to preserve against the relaxations. It should be noted that long-wavelength electromagnetic waves, as considered in this work, cannot provide momentum transfer of inter-valley transitions.

[1] F. Al-Dirini, F. M. Hossain, M. A. Mohammed, A. Nirmalathas, and E. Skafidas, Scientific reports **5**, 14815 (2015).
[2] M. Tahir and U. Schwingenschlögl, Scientific reports **3**, 1 (2013).
[3] F. Matusalem, M. Marques, L. K. Teles, and F. Bechstedt, Phys. Rev. B **92**, 045436 (2015).
[4] G. G. Guzmán-Verri and L. C. Lew Yan Voon, Phys. Rev. B **76**, 075131 (2007).
[5] S. Cahangirov, M. Audiffred, P. Tang, A. Iacomino, W. Duan, G. Merino, and A. Rubio, Phys. Rev. B **88**, 035432 (2013).
[6] E. Durgun, S. Tongay, and S. Ciraci, Phys. Rev. B **72**, 075420 (2005).
[7] M. Ezawa, New Journal of Physics **14**, 033003 (2012).
[8] H. Min, J. E. Hill, N. A. Sinitsyn, B. R. Sahu, L. Kleinman, and A. H. MacDonald, Phys. Rev. B **74**, 165310 (2006).
[9] M. Ezawa, Phys. Rev. B **86**, 161407 (2012).
[10] M. Ezawa, Phys. Rev. Lett. **110**, 026603 (2013).
[11] M. Ezawa, Journal of the Physical Society of Japan **84**, 121003 (2015).
[12] L. L. Tao and E. Y. Tsymbal, Phys. Rev. B **100**, 161110 (2019).
[13] F. Zhai, X. Zhao, K. Chang, and H. Q. Xu, Phys. Rev. B **82**, 115442 (2010).
[14] D. Xiao, W. Yao, and Q. Niu, Phys. Rev. Lett. **99**, 236809 (2007).
[15] W. Yao, D. Xiao, and Q. Niu, Phys. Rev. B **77**, 235406 (2008).
[16] K. Chan, Physics Letters A **382**, 534 (2018).
